# In Vitro and In Vivo Genotoxicity of Polystyrene Microplastics: Evaluation of a Possible Synergistic Action with Bisphenol A

**DOI:** 10.3390/jox14040079

**Published:** 2024-10-08

**Authors:** Alfredo Santovito, Mattia Lambertini, Alessandro Nota

**Affiliations:** 1 Department of Life Sciences and Systems Biology, University of Turin, Via Accademia Albertina 13, 10123 Torino, Italy; 2 Department of Chemistry, University of Turin, Via P. Giuria 7, 10125 Torino, Italy; mattia.lambertini@edu.unito.it; 3 Department of Biology and Biotechnology, University of Pavia, Via Ferrata 9, 27100 Pavia, Italy; alessandro.nota@conted.ox.ac.uk

**Keywords:** micronuclei, genotoxicity, microplastics, polystyrene, lymphocytes, hemocytes, *Lymnaea stagnalis*

## Abstract

The ubiquitous presence of plastics represents a global threat for all ecosystems and human health. In this study, we evaluated, in vitro and in vivo, the genotoxic potential of different concentrations of polystyrene microplastics (PS-MPs) and their possible synergistic interactions with bisphenol-A (BPA). For the in vitro and the in vivo assays, we used human lymphocytes and hemocytes from *Lymnaea stagnalis*, respectively. The genomic damage was evaluated by the micronucleus assay, and differences in eggs laid and growth of *L. stagnalis* were also evaluated. In human lymphocytes, PS-MPs alone at the concentration of 200 μg/mL and in association with BPA 0.100 µg/mL significantly increased the frequencies of micronuclei and nuclear buds, indicating a possible in vitro genotoxic additive action of these two compounds. Vice versa, PS-MPs did not result in genotoxicity in hemocytes. Our results indicated that PS-MPs have genotoxic properties only in vitro and at a concentration of 200 µg/mL; moreover, this compound could intensify the genomic damage when tested with BPA, indicating possible cumulative effects. Finally, PS significantly reduced the growth and the number of laid eggs in *L. stagnalis.*

## 1. Introduction

Due to a massive plastic production, quantifiable at more than 300 million tons per year [1], plastic debris is widespread and represents a threat to all ecosystems. In the different environments, this amount of plastic is subjected to abiotic degradation, with consequent formation of fragments of microplastics (MPs, diameter from 1 μm to 500 μm) and, secondarily, of nanoplastics (NPs, diameter, <100 nm). The wastewater treatment plants are not fully capable of trapping them through traditional filtration systems, and, consequently, most of these plastic particles remain in the environment, reaching marine and freshwater environments that represent their final acceptors [2,3].

MPs and NPs are able to enter living organisms and, due to their persistent nature, to accumulate in various organs and tissues [4,5].

In humans, MPs have been detected in many biological fluids and organs [6,7,8,9], as well as in the tumor tissue of patients with colorectal adenocarcinoma, indicating their possible association with this type of cancer [10]. At the cellular level, several studies have shown that MPs, due to their ability to penetrate and accumulate in cells, can induce inflammation, cytotoxicity, oxidative stress, and genotoxicity [11,12,13,14].

**Figure 1:**
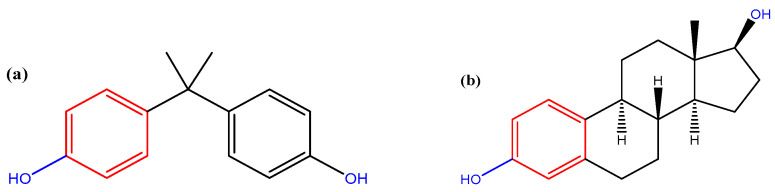
(**a**) Bisphenol A (BPA) and (**b**) estradiol; the molecular structure similarity between the two molecules allows them to interact with the same estrogen receptors (ER) [27]. The functional (phenolic) groups that give the xenobiotic BPA its similarity to estradiol in terms of interaction with the active site of the ER are highlighted in red (H-bond type interaction) and blue (π–π interaction).

Among the plastic polymers, polystyrene (PS) is one of the most produced and commonly present in the environment [15]. Because of its low cost and useful chemical-physical properties, PS has been widely used in the production and packaging of food, in electronics, in the automotive sector, and in many other sectors, including biomedicine, as part of diagnostic components and medical devices [16]. However, due to the massive production and its extreme resistance to being degraded [17], the PS micro- and nanoplastics are accumulating in all the ecosystems, causing great environmental concern [18]. Due to their small size, MPs can be easily confounded as food particles and ingested by many marine organisms, including zooplankton [19], bivalves [20], fish [21], crayfish [22], and mammals [23,24]. From a human health perspective, PS particles were found able to enter the human body, interact with blood and the lymphatic system, thereby reaching other organs and inducing oxidative stress and genomic damage [15]. BPA is a xenobiotic with a chemical structure similar to estrogen hormones (Figure 1); due to this structural similarity, it can bind to estrogen receptors (ER) on cells, mimicking the effects of its counterpart. This can lead to the activation of ER even in the absence of the hormone itself, causing an altered biological response and the disruption of the endocrine system [25,26].

The cytotoxicity due to NPs/MPs and bisphenol-A (BPA) can be possibly explained by a brief overview of their interaction with the metabolic pathways of animal cells. NPs/MPs, which are water-insoluble nano- and micromaterials, have both a mechanical and chemically harmful action. The physical presence of NPs/MPs within the cell can cause structural disruption in the mitochondrial and endoplasmatic reticulum membranes, leading to leakage of ions (perturbation of calcium homeostasis) and loss of membrane potential, which is crucial for proper mitochondrial functioning [28,29]. Furthermore, NPs/MPs can interfere with the normal functioning of the electron transport chain by a direct interaction with the electron transport protein; capturing electrons, NPs/MPs prevent O_2_ (the final electron acceptor) to be fully reduced to H_2_O, resulting in an excess of ROS production that is closely linked to oxidative stress and cellular disfunction [30]. Finally, NPs/MPs can further increase ROS production by acting as heterogenous catalysts in redox reactions since they are characterized by a carbon-based skeleton, functional groups, and even metals absorbed on surface [31].

**Figure 2:**
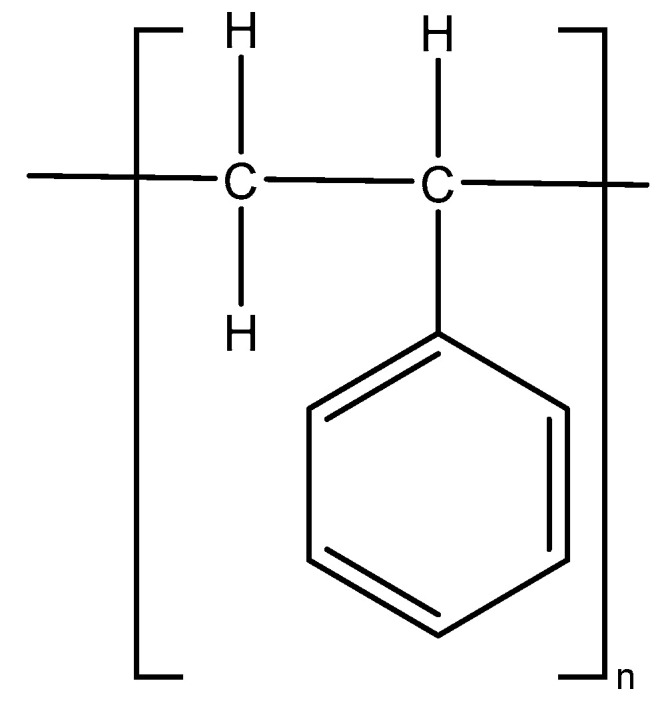
Molecular formula of the repeating unit of polystyrene.

Microplastics exhibit a high propensity for adsorbing diverse contaminants (metals and organic compounds) present in the environment [32,33,34,35,36]. Focus on the chemical structure of the polymer and xenobiotic allows to elucidate the way in which they interact. The interaction between BPA and PS-MPs, finely described with computational calculations and experimental analyses, involves physisorption, thus van der Waals forces [37,38,39]. Indeed, the greatest contributions are made by π–π stacking interaction, in which the π-electrons from the benzene rings in BPA (Figure 1) and PS (Figure 2) attract each other, electrostatic and hydrophobic interactions [39,40], and dispersion forces. It is important to emphasize that PS-MPs can change their molecular structure over time due to oxygen exposure and thermal and UV degradation. Consequently, a change in the atomic and structural composition corresponds to a change in the interaction mechanism. Evidence of that, the enhanced degree of oxidation increases the number of carbonyl groups, resulting in a disruption of aromatic rings; consequently, the π–π and hydrophobic interactions decrease, favoring polar bonding with BPA and providing changes in the (still non-covalent) reaction mechanism [32,38].

PS-MPs therefore might act as a sink of BPA that can be released through a desorption process once MPs enter inside the cell, possibly resulting in an exacerbated genotoxic effect due to the contemporary action of the two compounds. It is important to highlight that the physisorption mechanism is a crucial aspect for synergistic effects existence. Indeed, its intrinsically reversible nature allows pollutants to desorb from the MP surface and so potentially exert their toxic effect on cells. On the contrary, a chemisorption mechanism would imply a covalent bond to the MP surface. The bioavailability of contaminants is therefore suppressed; they still modify MP properties, possibly affecting its toxicity, but they are not able to take part in biologic pathways because of their irreversible bond (for a complete review on case history, see Huang et al., 2021) [41].

Expanding the discussion to other types of chemical actors, it has been observed that MPs and NPs can bond and/or act as carriers for other pollutants, promoting their bioaccumulation through the food chain [3,42,43,44]. Indeed, different in vivo studies showed the ability of MPs and NPs to transfer xenobiotic compounds to marine organisms, with their consequent accumulation in different tissues and organs, alteration of physiological parameters, and genotoxic effects [42,45,46,47,48].

Although the simultaneous exposure of MPs to other toxic agents represents one of the most important threats to aquatic organisms, conflicting data are available in the literature on the cellular and genotoxic effects resulting from the interaction of MPs with other pollutants [49,50].

The present study aimed to: (i) investigate the genotoxicity of PS-MPs at different concentrations in vitro by using human lymphocytes and in vivo by using *Lymnaea stagnalis;* and (ii) to investigate, in the same in vitro and in vivo systems and with the same polystyrene particles, the possible genotoxic synergistic action of PS-MPs in a mixture with BPA at a concentration of 0.100 µg/mL, whose genotoxic action is known [51]. Indeed, it is known that the absorption of chemical pollutants into plastic particles can increase the adverse effects of the plastic or the pollutants considered [41,52].

Genotoxicity was evaluated by the micronucleus (MN) assay conducted on both human lymphocytes and *L. stagnalis* hemocytes. Micronuclei originate from acentric chromosome fragments or whole chromosomes that fail to segregate correctly during the mitotic division, appearing as small additional nuclei in the cytoplasm of interphase cells. They represent both the clastogenic damage due to chromosome/chromatid breaks induced by xenobiotic compounds and the aneugenic damage due to agents that interfere with the mitotic apparatus, thus leading to the missegregation of whole chromatids or chromosomes during mitosis. Furthermore, chromosomal instability has been measured by evaluating nuclear buds (NBUDs), which represent the elimination process of amplified DNA and excess chromosomes from aneuploid cells [53,54,55].

In addition, the possible adverse effects of PS-MPs, alone and in combination with BPA, were also studied on two life-history traits of *L. stagnalis*: the number of laid eggs and the body growth.

## 2. Materials and Methods

### 2.1. Chemicals and Media

Gibco RPMI-1640 cell culture media supplemented with L-glutamine, fetal calf serum, phytohemagglutinin (PHA), and antibiotics were purchased from Invitrogen-Life Technologies, Milan, Italy. BPA, cytochalasin-B, mitomycin-C (MMC), Giemsa stain solution, potassium chloride (KCl), and Sörensen buffer were obtained from Merck S.p.A., Milan, Italy. Methanol, acetic acid, and conventional microscope slides were purchased from Carlo Erba Reagenti, Milan, Italy. Vacutainer blood collection tubes were from Terumo Europe, Rome, Italy. Polystyrene microplastic particles (PS-MP, product number: 89904) with analytical standard size of 1.0 μm, particle specific gravity of 1.05 g/cm^3^, and solid content of 10% were supplied by Merck S.p.A. (Milan, Italy) as an aqueous suspension (520 mg/L).

The chemistry of the tap water used for *L. stagnalis* experiments was as follows: pH: 7.3; dry residue at 180 °C: 313 mg/L; calcium: 71 mg/L; magnesium: 13 mg/L; ammonium: <0.05 mg/L; chlorides: 17 mg/L; sulphates: 35 mg/L; potassium: 2 mg/L; sodium: <10 mg/L; arsenic: <1 mg/L; bicarbonates: 238 mg/L; free residual chlorine: 0.1 mg/L; fluorides: <0.1 mg/L; nitrates: 21 mg/L; nitrites: <0.05 mg/L; manganese: <1 µg/L; BPs and MPs: absent.

The selected polystyrene is a monodispersed aqueous solution with a density of 0.520 g/L and a diameter of 1.0 μm. This PS diameter was selected because it is borderline between nano- and microplastics and because of its high environmental interest due to the fact that zooplankton species are not able to distinguish it from phytoplankton during the normal feeding and swimming activities [3].

Polystyrene was used at concentrations of 200, 100, 50, and 25 µg/mL. These concentrations were chosen to investigate potential synergistic effects with BPA, focusing on non-lethal concentrations that are lower than those used in other studies [1], and avoiding lethal doses that might mask any combined effect.

### 2.2. Subjects for In Vitro Experiments

Human lymphocytes were chosen as the in vitro model because blood is one of the main tissues that MPs can interact with [12,51]. Peripheral venous blood was collected from 20 healthy female subjects (mean age ± S.D., 22.150 ± 1.565, range 20–25 years). This age range was selected because the frequency of MNi can increase with age [56]. To minimize potential biases related to age, only young adults were involved. The subjects involved in the study received a questionnaire that included questions related to lifestyle factors. Only individuals who met the following criteria were included in the study: non-smokers, non-alcohol consumers, no recent drug therapy, or exposure to mutagenic substances or radiations. Informed consent was obtained from all blood donors. The study was approved by the University of Turin ethics committee (protocol number 0574348, date 18 October 2023) and was performed in accordance with the ethical standards laid down in the 2013 Declaration of Helsinki.

### 2.3. Blood Sample Collection, Lymphocyte Cultures and Cytokinesis-Block Micronucleus Assay

Blood samples were obtained by venipuncture and collected in heparinized tubes for genotoxicity testing. All blood samples were coded, cooled (4 °C), and processed within two hours after collection. Heparinized venous blood (0.3 mL) was cultured in 25 cm^2^ flasks in 6 mL of RPMI-1640 medium supplemented with 20% fetal calf serum (FCS), 2% of the mitogenic agent PHA, L-glutamine (2 mM), and antibiotics (100 IU/mL penicillin, and 100 μg/mL streptomycin). In a humidified atmosphere, the cultures were incubated for 72 h at 37 °C, under 5% CO_2_. After 24 h of incubation, PS-MPs were added to the pure cultures to final concentrations of 200, 100, 50, and 25 µg/mL and in combination with 0.100 µg/mL of BPA at the same concentrations. Moreover, two control cultures were prepared: (1) positive control by adding only MMC (final concentration 0.1 µg/mL culture); (2) negative control culture without polystyrene microplastics. After 44 h of incubation, cytochalasin-B was added to the cultures at a 6 µg/mL concentration to block the cytokinesis (Table 1).

After 72 h of incubation at 37 °C, the cells were collected by centrifugation and treated for 10 min with a pre-warmed mild hypotonic solution (75 mM KCl). After centrifugation and removal of the supernatant, the cells were fixed with a fresh mixture of methanol/acetic acid (3:1 *v*/*v*). The treatment with the fixative was repeated three times. Finally, the supernatant was discarded, and the pellet, dissolved in a minimal volume of fixative, was seeded on the slides to detect MNi and other nuclear anomalies by conventional staining with 5% Giemsa (pH 6.8) prepared in Sörensen buffer.

Microscope analysis was performed at 100× magnification on a light microscope (Dialux 20, Leica, Germany). MNi were scored in 1000 binucleated lymphocytes with well-preserved cytoplasm per subject per concentration (11,000 binucleated cells observed per subject per tested concentration, for a total of 220.000 cells analyzed), following the established criteria for MNi evaluation: (i) being morphologically identical to the nucleus and with the same staining, but with a diameter ranging from 1/16 to 1/3; (ii) being non-refractile; and (iii) lacking overlaps or connections to the main nucleus [53,54]. NBUDs are evaginations of the main nucleus and represent a biomarker of elimination of amplified DNA and/or DNA repair complexes. They are characterized by having the same morphology as an MN, but, unlike MN, they are linked to the nucleus by a narrow or wide stalk of nucleoplasmic material depending on the stage of the budding process [55,57]. 1000 lymphocytes per donor per concentration were scored to evaluate the cytokinesis-block proliferation index (CBPI) calculated according to the following formula: [1 × N1] + [2 × N2] + [3 × (N3 + N4)]/N, where N1–N4 represents the number of cells with 1–4 nuclei, respectively, and N is the total number of cells scored.

### 2.4. Lymnaea stagnalis

*L. stagnalis* was selected as a model organism due to its sensitivity as a non-target primary consumer in the trophic chain and to its high reproductive capability and relatively short lifespan. Moreover, an interesting advantage that this animal provides is the possibility to evaluate the influence of environmental xenobiotics on both its hemocytes and on some physiological parameters (egg production, growth) without killing it [58].

*L. stagnalis* individuals are simultaneous hermaphrodites, with a lifespan of about two years and a sexual maturity that occurs within three months after egg hatching [59]. *L. stagnalis* can be easily maintained in laboratory conditions because of its capacity to tolerate a wide range of temperatures, from about 0 °C to 26–28 °C, and pH values from 6 to 8.5. It is an omnivorous species and feeds mainly on algae and other plants [60]. *L. stagnalis* individuals involved in the study came from our parasite-free laboratory culture and were reared in the same water and feeding conditions. In order to avoid any confounding factor involving incomplete sexual maturity, reproductively mature individuals capable of producing eggs and with a shell length (i.e., distance from the apex to the farthest point on the outer margin of the aperture of the shell, following the central axis) ≥20 mm were randomly selected; this was done to exclude incomplete sexual maturity or inability to lay eggs. The experiment was conducted using 10 L containers filled with 6 L of tap water.

To test the in vivo genotoxic effects of the different concentrations of PS-MPs alone and in combination with 0.100 μg/mL of BPA, ten groups were randomly formed (Table 2).

In order to obtain the final concentrations of 200, 100, 50, and 25 µg/mL, a total of 10.5, 5.25, 2.625, and 1.3125 mL of PS were respectively dissolved in 6 L of water. The negative control was represented by water without PS.

In order to exclude any effect of biological variability due to individual-specific responses to the changing water conditions, all subjects involved in the study were reared under uniform conditions (i.e., tap water, temperature range between 18 and 22 °C, salad as primary source of food) and were kept under the same light/dark regime. During the 4-week study period, chosen to be in line with previous research [59,61,62], water, food, and BP concentrations were renewed twice a week for each group. The number of eggs laid was recorded each week; changes in shell growth (length in mm) were measured at the beginning and at the end of the experiment.

### 2.5. Micronuclei Assay on Hemocytes from Lymnaea stagnalis

After 4 weeks of xenobiotic exposure, hemolymph was collected by stimulating its release by means of prodding the foot of the animal with a micropipette. Five hundred microliters of hemolymph per subject was collected and distributed onto clear microscope slides. The cells were then fixed by adding several drops of methanol/acetic acid solution in a 3:1 ratio. The slides were dried and the cells stained for 10 min by a conventional staining method using 5% Giemsa (pH 6.8) prepared in Sörensen buffer. The slides were washed with distilled water and dried; after positioning the cover glass, the cells were observed under a Leica Dialux 20 light microscope (magnification 1000×). 1000 hemocytes with intact nuclear and cellular membranes were analyzed per subject per concentration, and the number of MNi and NBUDs was scored.

### 2.6. Statistical Analysis

Data normality and data distribution were tested both graphically and with the Shapiro–Wilk test. Since the CBPI and growth values had a normal distribution but did not show homoschedasticity (tested with the Levene test), differences for these variables were tested with a one-way ANOVA with the approximate method of Welch for unequal variances; post hoc analyses were performed using the Games–Howell test (rstatix package). Micronuclei and nuclear buds were not normally distributed in the four groups: since data on lymphocytes consisted of repeated measures on the same subjects, we applied the Friedman test (tidyverse package) and the Conover post hoc test (PMCMRplus package) to compare aberrations among the groups, while we used the Kruskal–Wallis test and the Dunn test as post hoc for the hemocytes. Differences in terms of the number of *L. stagnalis* laid eggs were tested with the chi-square test.

All statistical analyses were performed with R 4.3.2 (R core team, Vienna, Austria) and the Rstudio interface (RStudio Team, Boston, MA, USA). Graphs were created with GraphPad Prism 8 and R as well [63]. Statistical significance was indicated as * *p* < 0.05, ** *p* < 0.01, and ** *p* < 0.001.

## 3. Results

### 3.1. Lymphocytes

**Figure 3:**
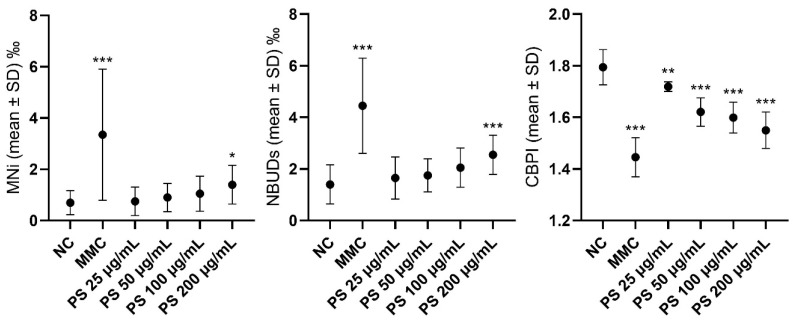
Graphs highlighting the differences in the levels of micronuclei, nuclear buds, and CBPI value in lymphocytes exposed to different concentrations of PS. CBPI = cytokinesis-block proliferation index; MMC = mitomycin C (positive control); MNI = micronuclei; NBUDs = nuclear buds; NC = negative control; PS = polystyrene. *** *p* < 0.001, ** *p* < 0.01, * *p* = 0.005, with respect to negative control.

In Figure 3, differences in the levels of MNi, NBUDs, and CBPI values in lymphocytes exposed to different concentrations of PS alone and in combination with BSA at the concentration of 0.100 µg/mL are highlighted. The analytical data are reported in Supplementary Material. PS significantly increased the MNI and NBUD frequencies only at concentrations of 200 μg/mL. Vice versa, a significant reduction in the CBPI was observed when the NC was compared with the positive control (MMC) and with all tested PS concentrations.

**Figure 4:**
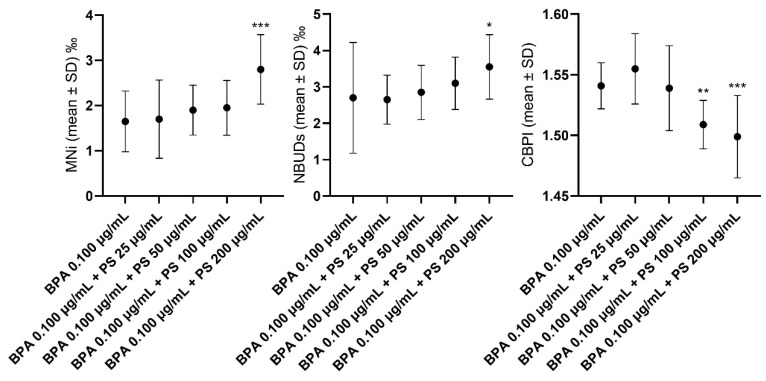
Graphs highlighting the differences in the levels of micronuclei, nuclear buds, and CBPI values in lymphocytes exposed to different concentrations of PS in combination with BPA at the concentration of 0.100 μg/mL, with respect to the latter alone. BPA = bisphenol A; CBPI = cytokinesis-block proliferation index; MNi = micronuclei; NBUDs = nuclear buds; PS = polystyrene. *** *p* < 0.001, ** *p* < 0.01, * *p* = 0.005, with respect to BPA 0.100 µg/mL.

In Figure 4, differences in the levels of MNi, NBUDs, and CBPI values in lymphocytes exposed to BPA 0.100 µg/mL alone and in combination with different concentrations of PS are highlighted. The analytical data are reported in SM1. PS at the concentration of 200 μg/mL, in association with BPA at the concentration of 0.100 μg/mL, significantly increased the MNI and NBUDs frequencies with respect to BPA 0.100 μg/mL tested alone. Compared to the latter, a significant reduction in the CBPI value was observed when BPA 0.100 µg/mL concentrated was associated with PS at concentrations of 100 and 200 µg/mL.

### 3.2. Hemocytes

**Figure 5:**
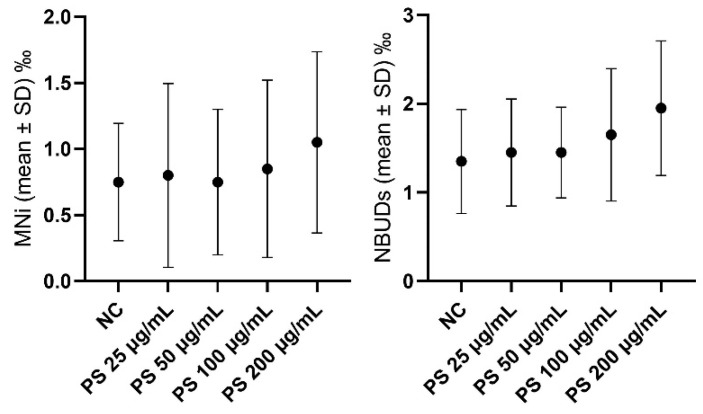
Frequencies of MNi and NBUDs in hemocytes of *L. stagnalis* exposed to different concentrations of PS. MNi = micronuclei; NBUDs = nuclear Buds; NC = negative control.

In Figure 5 and SM1, differences in the levels of MNi and NBUDs in hemocytes of *L. stagnalis* exposed to different concentrations of PS are highlighted. With respect to the negative control, PS at all tested concentrations did not result in genotoxicity in hemocytes in terms of increased levels of MNi or NBUDs.

**Figure 6:**
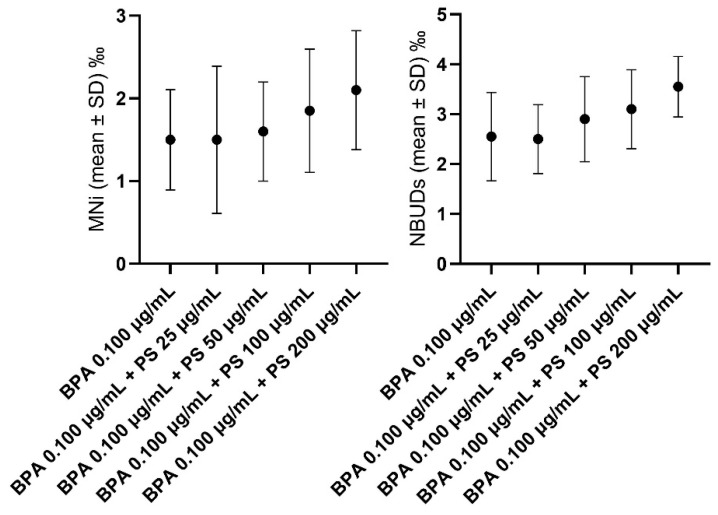
Frequencies of MNi and NBUDs in hemocytes of *L. stagnalis* exposed to BPA 0.100 to µg/mL and to BPA 0.100 to µg/mL associated with the different concentrations of PS. BPA = bisphenol-A; MNi = micronuclei; NBUDs = nuclear buds; PS = polystyrene.

In Figure 6 and SM1, differences in the levels of MNi and NBUDs in hemocytes of *L. stagnalis* exposed to different concentrations of PS alone (A) and associated with BPA 0.100 µg/mL concentrated (B) are highlighted. With respect to the negative control, PS at all tested concentrations did not result in genotoxicity in hemocytes in terms of increased levels of MNi or NBUDs (A). We observed the same result when the genotoxicity of PS at all concentrations and associated with BPA 0.100 to µg/mL was compared to that of BPA 0.100 µg/mL tested alone (B).

**Figure 7:**
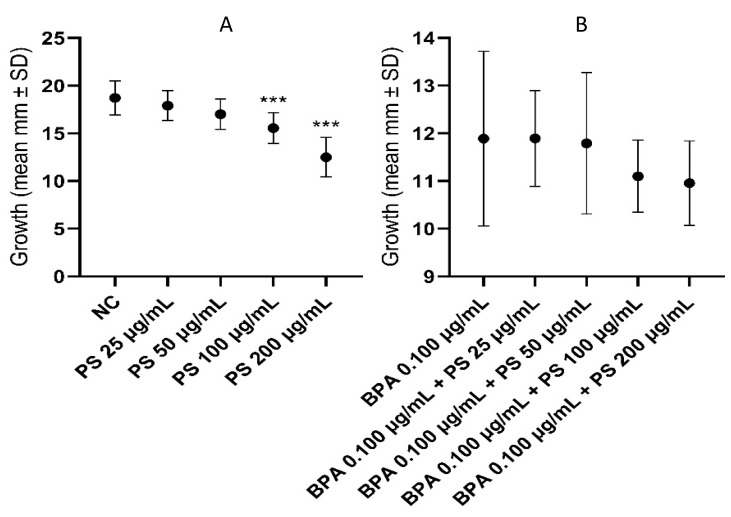
Differences in shell growth in *L. stagnalis* individuals exposed to different concentrations of PS tested alone (**A**) and in association with BPA 0.100 µg/mL (**B**). BPA = bisphenol-A; PS = polystyrene. *** *p* < 0.001.

In Figure 7 and SM1, differences in shell growth of *L. stagnalis* exposed to different concentrations of PS alone (A) and associated with BPA 0.100 µg/mL are shown (B). With respect to the negative control, a significant shell growth reduction was observed in subjects exposed to 100 and 200 μg/mL of PS (A). Vice versa, PS at all concentrations associated with BPA 0.100 to µg/mL did not induce any significant difference in shell growth when compared to BPA 0.100 to µg/mL tested alone (B).

**Figure 8:**
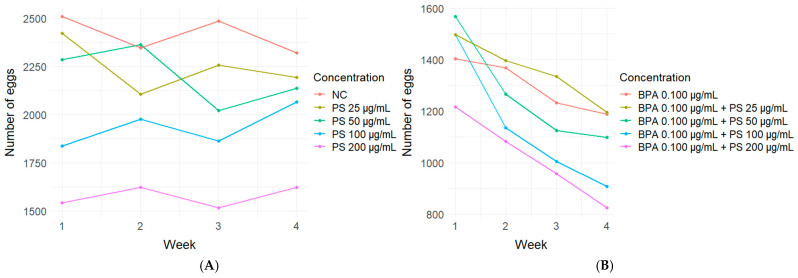
Trend of the number of laid eggs per week for PS at all tested concentration tested alone (**A**) and in association with BPA 0.100 µg/mL (**B**).

In Figure 8, the trend of the number of eggs laid per week, for each PS concentration (A) and for each PS concentration in association with BPA 0.100 µg/mL (B), is shown. In SM1, the analytical data are reported. PS at all concentrations induced a significant reduction in the number of laid eggs with respect to the negative control (A). Similarly, with respect to BPA 0.100 µg/mL tested alone, when PS was tested in association with BPA 0.100 µg/mL, it induced a significant reduction in egg production at all concentrations, with the exception of 25 µg/mL.

## 4. Discussion

The ubiquitous presence of plastics, and in particular of MPs and NPs, represents a global threat for all ecosystems and for human health. Among plastic detritus, PS-MPs are one of the most represented in the environment. These small particles are able to enter the body of all organisms and to reach, by blood and the lymphatic systems, all organs [1].

In the present study, we aimed to investigate the genotoxicity of different concentrations of PS-MPs with a diameter of 1.0 μm. Although it is known that the absorption of chemical pollutants into plastic particles can increase the adverse effects of the plastic or the pollutants considered, to date, the synergistic interactions between MPs and endocrine disruptor compounds are still poorly explored [43,52]. For this reason, the second aim of the study was to evaluate, in human lymphocytes and *L. stagnalis* hemocytes, the ability of PS-MPs to amplify the genotoxic effects of the BPA.

### 4.1. In Vitro Experiment

For the in vitro study, we used human peripheral blood lymphocytes as a cell system in order to evaluate the genotoxicity of PS-MPs tested alone at different concentrations or in association with BPA 0.100 µg/mL. We investigated the genotoxic effects by the MNi assay, which allows the evaluation of MNi and NBUD frequencies and the replication capacity of the cells by the CBPI index. We observed genotoxic effects of PS in terms of significantly increased frequencies of MNi and NBUDs, only at the highest concentration of 200 μg/mL. This result partially agrees with those obtained by Cortés et al. (2020) [64], who exposed Caco-2 cells to different concentrations of polystyrene nanoplastics, including the highest concentration of 200 µg/mL. The authors demonstrated that exposure to polystyrene nanoplastics was not able to induce significant increases in the frequency of micronuclei, even at the concentration of 200 µg/mL that in our work resulted in genotoxicity. Similarly, Hwang et al. (2022) [65] observed cytotoxic properties of PS in human dermal fibroblasts and peripheral blood lymphocytes only at the extremely high concentrations of 500 µg/mL, more than double the highest concentration we used. The different susceptibility to xenobiotic compounds of the cells used in these different studies could be a possible explanation of these partially discrepant results.

The situation is similar when considering the genotoxicity of PS nanoparticles. Indeed, small-sized polystyrene nanoplastics were able to cause cytotoxic and genotoxic effects in peripheral human blood cells, in terms of increased frequencies of MNi and NBUDs, and in a concentration-dependent manner [1,14].

MPs may also act as possible carriers of xenobiotic hydrophobic compounds, such as endocrine-disrupting chemicals [66,67]. In our work, with respect to BPA at the concentration of 0.100 μg/mL, we observed that the binary association of BPA and PS-MPs resulted in a significant increase in genotoxicity when PS-MPs were present at the highest concentration of 200 μg/mL, indicating a possible cumulative genotoxic effect of BPA and PS when this last is administered at the minimal concentration of 200 µg/mL. This might be alarming, as the ubiquitous presence of both MPs and BPA in the environment could be dangerous for both specifically exposed populations, such as industrial workers, and for residents of industrial areas who are at a high risk of exposure to several emerging pollutants.

A significant reduction in the CBPI was observed in culture treated with PS alone at all concentrations, indicating that PS, at all tested concentrations, negatively affects the replication capacity of the cells. Similar data were obtained by Inkielewicz-Stepniak et al. (2018) [68], who observed that polystyrene nanoparticles affected cell viability, and by Sarma et al. (2022) [1], who showed a reduction in the mitotic index in peripheral blood monocytes after exposure to PS nanoplastics, although at higher concentrations (500, 1000, and 2000 µg/mL) with respect to those tested in the present work. All these data seem to indicate an increase in the cytostasis induced by both PS-MPs and PS-NPs on human blood cells, evidencing the inhibitory role of PS-NP and PS-MPs on cellular proliferation. Finally, a significant reduction in CBPI was observed also when PS was tested at the concentrations of 100 and 200 µg/mL in association with BPA 0.100 µg/mL, confirming, also in this case, a cumulative action of these compounds in determining the reduction in the replicative capacity of the cells.

### 4.2. In Vivo Experiment

Published studies have highlighted that MPs and NPs can infiltrate the bodies of all organisms, penetrate biological membranes, and access organs, tissues, and cells [15,69]. As to the selected biological model, hemocytes represent the most important front of immune defense and, in toxicological studies, a suitable rapid screening tool for genotoxicity profiling of pollutants [58,70,71].

As a principal result of our work, PS alone did not result in genotoxicity in hemocytes at all tested concentrations, neither in terms of increased levels of MNi nor in terms of NBUDs. This reduced genotoxicity observed in vivo with respect to the in vitro approach could be probably explained by the capacity of organisms to better both metabolize xenobiotics and repair the DNA damage, with respect to a cellular system.

However, our results are in contrast with those found by other authors, who observed that PS-MPS were able to induce DNA damage in mussel hemocytes [72] and strand breaks in the hemocytes of clams [73], respectively. Moreover, Nugnes et al. (2022) in *Ceriodaphnia dubia* [3] and Ansoar-Rodríguez et al. (2015) in *Oreochromis niloticus* [74], observed that PS-MPs cause DNA damage at concentrations in the order of units and hundreds of μg/L, respectively. In both these last two cases, the authors explained the observed toxic effects by the increase in ROS, which are able to affect the genetic material. These contrasting results could also be justified by a possible different susceptibility of different organisms to environmental xenobiotics. *L. stagnalis* is a mollusk that inhabits ponds and, in some cases, highly polluted areas and, thus, probably evolved more efficient mechanisms of DNA repair due to exposure to high concentrations of pollutants.

Although some studies reported that the combined exposure to microplastic particles and drugs can increase the toxicity in aquatic organisms [75,76], in the present study we did not observe any increase in genotoxicity when PS at all concentrations was tested in associations with BPA at the concentration of 0.100 μg/mL.

This result is in contrast to results obtained by other authors. Indeed, in a previous study, the immunotoxicity and neurotoxicity caused by BPA were aggravated by the addition of MPs in the clam *Tegillarca granosa*, suggesting synergistic interactions between the two compounds [77]. Similarly, Zhou et al. (2020), exposing *Tegillarca granosa* to PS microplastic particles (500 nm) and veterinary antibiotics (oxytetracycline and florfenicol), observed that microplastic particles aggravated the bioaccumulation of these antibiotics, also suppressing the clam glutathione-S-transferase activity and other detoxification processes [78].

Combined exposure to PS nanoparticles (110 nm, 0.05 mg/L) and carbamazepine was also found to induce a significant increase in DNA damage and a downregulation in the expression of some genes in hemocytes of the mussel *Mytilus galloprovincialis* [79]. Furthermore, Sun and collaborators (2021) observed that microplastics may favor the accumulation of pesticides in earthworms, causing exacerbated oxidative damage and alterations of different metabolic pathways [80]. One possible explanation might be driven by the filtering and digging nature of such species, which may have predisposed them to greater contact with the MPs compared to the snail *L. stagnalis*. In contrast, *L. stagnalis*, being a surface grazer, may have less direct exposure to microplastics, limiting the extent of both plastics and other contaminants bioaccumulation. This reduced contact with microplastics could explain why no significant genotoxic or physiological effects were observed in our study: the biological characteristics of *L. stagnalis* may provide a natural barrier to the accumulation of contaminants, also contributing to the absence of synergistic effects in our findings. Another possible reason for the lack of significant synergistic effects could be the relatively low adsorption capacity of BPA by PS-MPs compared to other plastic polymers, such as thermoplastic polyurethane (TPU) and polyamide (PA) [81]. Although these data were known to us beforehand, we felt it was important to study the effects of PS since, unlike TPU and PA, it is one of the most common plastic polymers produced worldwide [82].

However, it is important to emphasize that even in other cases, the synergistic effects were not so evident. Nobre et al. (2020), in the oyster *Crassostrea brasiliana*, found a lower genotoxic effect after exposure to polyethylene (150–250 μm) and triclosan compared to the exposure to the only microplastic particles [83]. Probably, as explained by Birben et al. (2012), when organisms are stimulated by external factors, they generate free radicals as a defense mechanism, but, in this case, their antioxidant defense system is stimulated to remove these free radicals, protecting cells from oxidative damage [84]. The efficiency in removing free radicals could be different in different organisms, and this would explain the contrasting results of the literature.

Analyzing other physiological parameters, we found that, after 4 weeks of treatment, PS at the concentrations of 100 and 200 µg/mL caused a significant reduction in the growth rate, whereas a synergistic action with BPA 0.100 µg/mL was not observed. The latest data are in contrast with those reported by Luo et al. (2021), who showed that PS and imidacloprid combined in a chronic toxicity assay affected the growth of zebrafish [85]. Moreover, Zhu et al. (2019), combining different types of MPs (polystyrene, polyethylene, and polyvinyl chloride; 74 μm) and antibiotic drugs (triclosan), observed an antagonistic effect on growth inhibition in the algae *Skeletonema costatum* [86].

Finally, in the present study, an important effect was observed in terms of a significant reduction in laid eggs, both of PS alone at all concentrations and in association with BPA 0.100 µg/mL. These results are in line with those obtained by Nugnes et al. (2022), who demonstrated that PS-MPs were able to reduce the *C. dubia* offspring [87]. Similarly, Felten et al. (2020), in *Daphnia magna,* observed synergic adverse effects on the survival and fertility when polyethylene microplastic particles were combined with the pesticide deltamethrin [88].

## 5. Conclusions

In the present work, we evidenced in vitro genotoxic properties of PS-MPs when tested alone at the highest concentration of 200 µg/mL or in association with BPA, also in this case at the highest concentrations of 200 and 100 µg/mL. However, these effects were not replicated in the in vivo experiment.

While the additive genotoxic effects of PS and BPA in vitro raise concerns about the potential risks of microplastics when combined with endocrine disruptors, this interactive toxicity was not observed in our model organism. This discrepancy highlights a crucial research gap in understanding species-specific responses to the combined exposure to plastics and other contaminants.

In general, microplastics are known carriers of organic pollutants, posing severe threats to the environment and human health. However, the mechanisms by which these particles interact with pollutants, particularly in aquatic organisms, remain largely underexplored. Future research will need to focus on understanding the factors that affect the variability in bioaccumulation and toxicity across different taxa, particularly non-filter feeders like *L. stagnalis*, where bioaccumulation may be less pronounced. Investigating the long-term effects of exposure to microplastics and their associated pollutants is also essential, as available literature is mainly limited to short-term exposures.

In aquatic ecosystems, the impact of combined exposures to microplastic particles and drugs or pesticides raises several concerns. Addressing these research gaps will help to develop a more complete understanding of the risks posed by microplastics and guide environmental policies to mitigate their impacts.

## Figures and Tables

**Table 1:** Experimental set-up for the in vitro human lymphocyte assay. Number of tested subjects: 20 for group; exposure time: 72 h.

Group	Treatment
Negative control	0.000 μg/mL of fresh water
Positive control	0.100 µg/mL of mytomicin-C
Culture 1	0.100 μg/mL of BPA
Culture 2	200 μg/mL of PS
Culture 3	100 μg/mL of PS
Culture 4	50 μg/mL of PS
Culture 5	25 μg/mL of PS
Culture 6	200 μg/mL of PS + 0.100 μg/mL of BPA
Culture 7	100 μg/mL of PS + 0.100 μg/mL of BPA
Culture 8	50 μg/mL of PS + 0.100 μg/mL of BPA
Culture 9	25 μg/mL of PS + 0.100 μg/mL of BPA

**Table 2:** Experimental set-up for *L. stagnalis*. Exposure time: four weeks.

Group—20 Individuals per Container	Treatment
Controls	0.000 μg/mL of fresh water
Group 1	0.100 μg/mL of BPA
Group 2	200 μg/mL of PS
Group 3	100 μg/mL of PS
Group 4	50 μg/mL of PS
Group 5	25 μg/mL of PS
Group 6	200 μg/mL of PS + 0.100 μg/mL of BPA
Group 7	100 μg/mL of PS + 0.100 μg/mL of BPA
Group 8	50 μg/mL of PS + 0.100 μg/mL of BPA
Group 9	25 μg/mL of PS + 0.100 μg/mL of BPA

## Data Availability

Analytical data are reported in Supplementary Materials. Statistical analysis results and other data generated during the course of this study are available upon request.

## Supplementary Materials

The following supporting information can be downloaded at: https://www.mdpi.com/article/10.3390/jox14040079/s1.
